# Potential added value of an AI software with prediction of malignancy for the management of incidental lung nodules

**DOI:** 10.1016/j.redii.2023.100031

**Published:** 2023-10-21

**Authors:** Bastien Michelin, Aïssam Labani, Pascal Bilbault, Catherine Roy, Mickaël Ohana

**Affiliations:** a Department of Diagnostic Imaging (Radio B), Hôpitaux universitaires de Strasbourg, Strasbourg 67000, France; b Emergency Department, Hpitaux universitaires de Strasbourg, Strasbourg 67000, France

**Keywords:** Lung nodule, Lung cancer, Artificial intelligence, Radiomics, Deep learning

## Abstract

**Purpose:**

To determine the impact of an artificial intelligence software predicting malignancy in the management of incidentally discovered lung nodules.

**Materials and methods:**

In this retrospective study, all lung nodules ≥ 6 mm and ≤ 30 mm incidentally discovered on emergency CT scans performed between June 1, 2017 and December 31, 2017 were assessed. Artificial intelligence software using deep learning algorithms was applied to determine their likelihood of malignancy: most likely benign (AI score < 50%), undetermined (AI score 50–75%) or probably malignant (AI score > 75%). Predictions were compared to two-year follow-up and Brock's model.

**Results:**

Ninety incidental pulmonary nodules in 83 patients were retrospectively included. 36 nodules were benign, 13 were malignant and 41 remained indeterminate at 2 years follow-up.

AI analysis was possible for 81/90 nodules. The 34 benign nodules had an AI score between 0.02% and 96.73% (mean = 48.05 ± 37.32), while the 11 malignant nodules had an AI score between 82.89% and 100% (mean = 93.9 ± 2.3). The diagnostic performance of the AI software for positive diagnosis of malignant nodules using a 75% malignancy threshold was: sensitivity = 100% [95% CI 72%-100%]; specificity = 55.8% [38–73]; PPV = 42.3% [23–63]; NPV = 100% [82–100]. With its apparent high NPV, the addition of an AI score to the initial CT could have avoided a guidelines-recommended follow-up in 50% of the benign pulmonary nodules (6/12 nodules).

**Conclusion:**

Artificial intelligence software using deep learning algorithms presents a strong NPV (100%, with a 95% CI 82–100), suggesting potential use for reducing the need for follow-up of nodules categorized as benign.

## 1 Introduction

Lung cancer is the leading cause of cancer death worldwide [1], with a prognosis depending on the stage of the disease at the time of diagnosis [2]. It frequently presents as a lung nodule on computed tomography (CT). With the increasing use of CT, the incidental discovery of a lung nodule is a common situation, with an estimated prevalence of incidental pulmonary nodules – i.e. detected fortuitously on scans performed for another reason - of 13% [3], [4], [5], [6], [7], [8], [9], [10]. While the majority of these incidental pulmonary nodules are benign, a significant number of them represent cancers, potentially curable since discovered at an early stage [[11], [12]]. The differentiation between benign and malignant pulmonary nodules is therefore essential, in order to identify and treat malignant lesions early, while avoiding invasive explorations – and associated risks – in benign lesions.

There are guidelines aiming to avoid over-medicalization of benign lesions. The updated 2017 Fleischner Society guidelines are the most commonly used and are essentially based on the size of the nodule and its morphology[13]. Mathematical prediction models also exist, such as Brock's multivariate model developed from the data of more than 80 000 patients, which calculates a probability of nodule malignancy by integrating clinical and radiological criteria [14]. This probability can be integrated into decision algorithms to define the most appropriate management, as in the recommendations of the British Thoracic Society[3]. However, these recommendations have limitations since it is difficult to predict the malignancy of a nodule from the initial scan alone, as the visual criteria of malignancy lack specificity [15]. Therefore, many indeterminate nodules require CT follow-up. These follow-up recommendations, which are sometimes restrictive, are not systematically applied and are insufficiently respected in current clinical practice [[16], [17]].

The recent boom in artificial intelligence has enabled the development of a new generation of software able to predict malignancy, which appears promising for evaluating the benign or malignant nature of a pulmonary nodule [18]. Based on machine-learning and deep learning models, it uses large datasets to achieve potential lesion characterization [[19], [20]]. However, the impact of this type of predictive software is yet unknown, particularly on the management of incidentally discovered pulmonary nodules.

The main objective of this study is therefore to assess the clinical impact of a software aiming to predict malignancy for the management of pulmonary nodules discovered incidentally on emergency CT scans.

The secondary objectives are to analyze the feasibility and performance of this type of software, and to compare them with a multivariate analysis model validated for clinical practice (Brock's model).

## 2 Materials and methods

This study has been approved by the ethics committee of our hospital (Hôpitaux universitaires de Strasbourg, France; IRB HUS-7311). Informed consent from patients was not required to conduct this retrospective study.

### 2.1 Study population

All patients who performed – in an emergency context – a CT scan that included the thorax between June 1st, 2017 and December 31st, 2017 were considered for retrospective inclusion in this work.

The radiology report of each examination was accessed by the same practitioner (BM, radiologist with 3 years of experience in medical imaging) for pre-selection. The patients were ultimately selected on the re-reading of their CT and the analysis of their electronic health records.

The inclusion criteria were:

- Presence of at least one pulmonary nodule, whatever its type (solid, mixed or ground glass)
- With a larger diameter ≥ 6 mm and < 30 mm.

The exclusion criteria were:

- nodules already known at the time of the examination;
- Multiple nodules (> 3)
- Fully calcified nodules;
- Nodules in patients with an active cancer or in remission for less than 5 years.

For each patient with up to 3 nodules meeting the inclusion and exclusion criteria, the following data were listed:

- Age;
- Sex;
- Smoking status (active smoker / weaned smoker / non-smoker).

### 2.2 Pulmonary nodule

#### 2.2.1 Image analysis

Chest CT were all acquired on a 320-row scanner (Aquilion® ONE Vision Edition, Canon Medical Systems) or a 64-row scanner (Discovery 750 CT, GE Healthcare) using helical acquisition. CT were reconstructed in lung window (width = 1500 Housfield unit [HU]; center = −700 HU) with a hard kernel and a slice thickness of 1 mm, using an iterative reconstruction algorithm. An injection of iodinated contrast media was or was not performed, depending on the indication for the examination. Whenever possible, the images were obtained in blocked apnea, arms raised above the head.

A single radiologist (BM) retrospectively analyzed all the included examinations and gathered for every included lung nodule:

- Its maximum diameter (in mm);
- Its lobar location (right upper lobe, middle lobe, right lower lobe, left upper lobe, left lower lobe)
- Its type (solid, mixed or ground-glass);
- Its border (irregular or regular);
- The presence or not of an underlying pulmonary emphysema;
- The potential recommendation for nodule management appearing in the report by the radiologist who initially read the CT.

#### 2.2.2 Follow-up

Electronic health records were consulted on April 31st, 2021, to know the evolution of all pulmonary nodules included.

A nodule was classified as malignant when definitive pathology evidence (biopsy or surgical excision) was available.

A nodule was considered benign:

- If it was stable or reduced in size at a minimum of 2 years follow-up;
- If it had completely disappeared on any follow-up examination;
- If the pathology (representative biopsy, surgery) was benign;
- Or if it presented all the characteristics of an intrapulmonary lymph node in accordance with the Fleischner 2017 recommendations (nodule <10 mm, triangular or ovoid, with a subpleural topography and below the level of the carina).

Indeterminate nodules were defined by:

- No follow-up or follow-up <2 years;
- And/or no pathology available;
- And/or the absence of the typical characteristics of an intra-pulmonary lymph node.

### 2.3 Artificial intelligence analysis

The anonymized DICOM data were sent to a dedicated local server for its analysis by an Artificial Intelligence software (InferRead CT Lung, Infervision, China). The software automatically detects and segments the pulmonary nodule(s) and specifies a score (“AI score”) between 0 and 100 to define the probability of malignancy for each of them.

This commercially available software uses deep learning algorithms to detect suspicious pulmonary nodules and automatically generate the probability of malignancy. In order to train the detection and classification models, thin-slice chest CT scans were retrospectively collected and anonymized from multiple Chinese hospitals from Beijing, Hubei, and Liaoning provinces. All included scans used for the detection model training were annotated by experienced radiologists using bounding boxes to indicate the presence of pulmonary nodules. Then, the available pathology reports of pulmonary nodules were also collected for developing the classification model. In total, approximately 20,000 chest scans, 300,000 lung nodules and 3000 to 4000 pathology reports were used for the development of the detection and classification model.

The pulmonary nodule detection function is based on a DenseNet model – in order to extract feature map - and a Faster RCNN model - to detect target regions. The detection model allows to generate bounding boxes indicating the presence of suspicious pulmonary nodules on CT scans. The classification model uses respectively U-Net and CapNets to capture low-level and high-level features. The output of the network is an indication of the probability of the nodule to be malignant. The detailed process of model development and model performance are described in previous studies [21].

In accordance with the manufacturer's recommendations, according to the score obtained, the results of the software were classified into 3 categories:

- AI score <50%: low risk. The nodule is classified as benign.
- AI score 50–75%: intermediate risk. The nodule is unclassified.
- AI score> 75%: high risk. The nodule is classified as malignant.

### 2.4 Assessment of the probability of malignancy according to Brock's model

The probability of malignancy was calculated for each nodule following Brock's model [14].

This multivariate analysis model integrates 9 independent clinical and radiological predictive criteria to define a probability of malignancy of a node. These criteria are age; sex; family history of lung cancer; emphysema; nodule size; numbers of nodes; spiculation; mixed density; localization in a superior lobe.

The nodules were then categorized into:

- Probably benign nodule (probability of malignancy according to Brock < 5%);
- Possibly malignant nodule (probability of malignancy according to Brock ≥ 5%).

### 2.5 Statistical analysis

Descriptive statistics were used to present results. Qualitative variables were presented as counts and percentages; quantitative variables were presented as mean ± standard deviation, with minimum and maximum limits. Fisher's exact test was used to compare qualitative variables between malignant nodules and benign nodules. Mann-Whitney test was used to compare quantitative variables. A threshold of 0.05 was considered as statistically significant.

The performance of the diagnostic software was evaluated by comparison with the final status of the nodule (benign or malignant). Sensitivity (Se), specificity (Sp), positive predictive value (PPV) and negative predictive value (NPV) were calculated, with exclusion of indeterminates nodules from the performance analysis.

## 3 Results

### 3.1 Study population

**Fig. 1:**
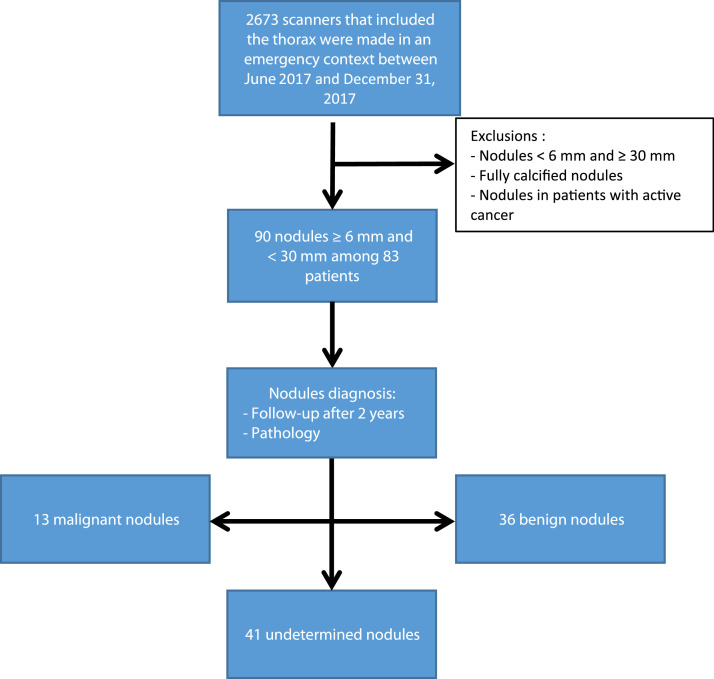
Pulmonary nodules flowchart.

Two thousand six hundred and seventy-three consecutive emergency CT which included the thorax were performed between June 1, 2017 and December 31, 2017. The retrospective analysis of all associated reports and images identified 83 patients totalizing 90 lung nodules that ultimately met the inclusion and exclusion criteria. A flowchart of the study is provided in Fig. 1.

**Fig. 2:**
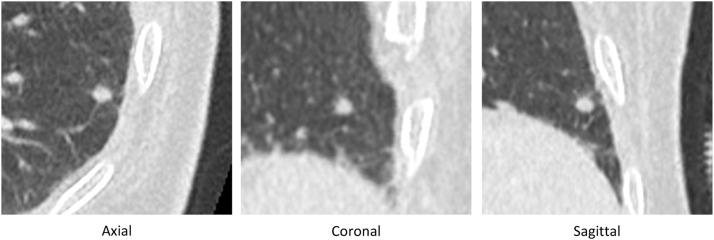
Example of a nodule which was confirmed benign (stability 2 years later), which was correctly categorized as benign by the artificial intelligence software (AI score = 1.12) (AI score between 50 and 75%).

**Fig. 3:**
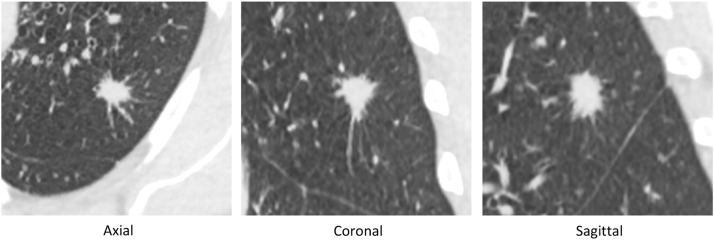
Example of a nodule which was confirmed malignant by histological analysis (squamous cell carcinoma), which was correctly categorized as malignant by the artificial intelligence software (AI score= 95.25).

**Fig. 4:**
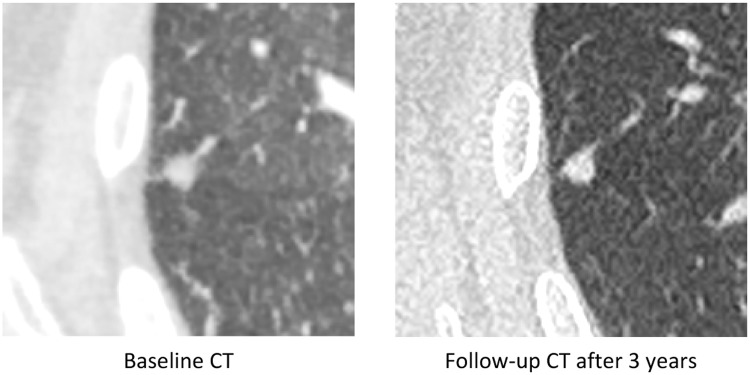
Example of a benign nodule of 8 mm of RLL in axial slices, which was subject to several follow-up CT (stability after 3 years). The nodule was already categorized as benign by the AI ​​software on the Baseline scanner (IA score <50%).

**Fig. 5:**
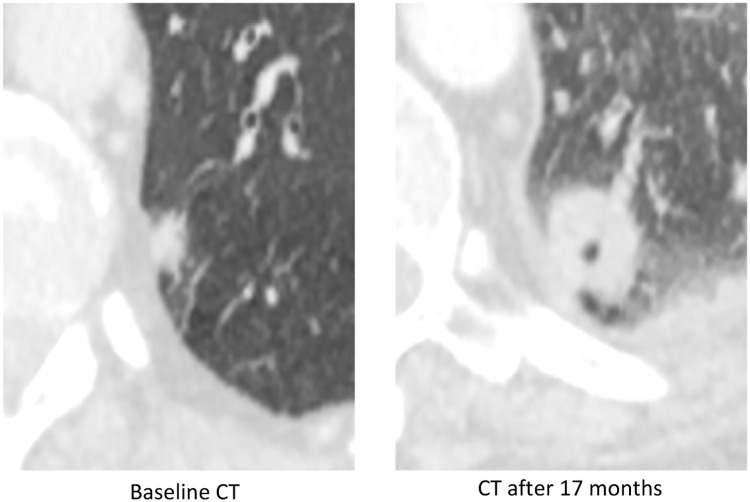
Example of a malignant nodule of 10 mm of the LLL in axial slices on the baseline scanner. A recommendation of follow-up after 3 months was made but was not implemented. A CT scan performed 17 months later out of context for suspected pulmonary embolism revealed a significant increase in the nodule, ultimately corresponding to an adenocarcinoma confirmed by lung biopsy. *The use of AI software could potentially have reduced this diagnostic delay by increasing the need for follow-up, given the high AI score of the nodule on the CT Baseline (IA Score = 94.52)*.

The demographic characteristics of the population were: 54 men and 29 women, an average age of 68.6 years (28 to 97); 31 patients were active smokers (37.4%), 27 were weaned smokers (32.5%) and 35 were non-smokers (42.1%) Fig. 2, Fig. 3, Fig. 4, Fig. 5.

### 3.2 Pulmonary nodules

The characteristics of the 90 nodules are listed in Table 1.

**Table 1:** Characteristics of pulmonary nodules

		Malignant nodules (*N* = 13)	Benign nodules (*N* = 36)	Undetermined nodules (*N* = 41)	*p* malignant vs benign	**Total (*N*** **=** **90)**
Size (mm)		13,9 ± 5.0 [8–25]	9.7 ± 4.8 [6–25]	11.3 ± 5.0 [6–26]	0.003°	11 ± 5.0 [6–26]
Contours	Regular	4 (30.8)	30 (83.3)	28 (68.3)	0.001±	62 (69)
	Irregular	9 (69.2)	6 (16.7)	13 (31.7)		28 (31)
Type	Solid	13 (100)	33 (91.7)	39 (95.1)	1±	85 (94.5)
	Ground Glass	0 (0)	1 (2.8)	1 (2.4)		2 (2.2)
	Mixed	0 (0)	2 (5.6)	1 (2.4)		3 (3.3)
Location	RUL	5 (38.4)	6 (16.7)	17 (41.5)	0.083±	28 (31.1)
	ML	0 (0)	8 (22.2)	6 (14.6)		14 (15.5)
	RLL	1 (7.7)	10 (27.8)	5 (12.2)		16 (17.8)
	LUL	3 (23.1)	4 (11.1)	9 (21.9)		16 (17.8)
	LLL	4 (30.8)	8 (22.2)	4 (9.8)		16 (17.8)
Emphysema	Presence	11 (84.6)	14 (38.9)	14 (34.1)	0.008±	39 (43.3)
	Absence	2 (15.4)	22 61.1)	27 (65.9)		51 (56.7)

° Fischer's exact test

± Mann Whitney test.

At least one follow-up CT was available for 41 out of 90 nodules (45%). If considering the longest follow-up only, the average follow-up on these 41 nodules was 18.4 months [1–41].

A management recommendation by the radiologist who initially read the examination was present in the initial report for 50 out of 90 nodules (56%).

At the end of the follow-up and analysis of electronic patient records, the nodules were classified as confirmed benign, confirmed malignant or undetermined:

- 36 nodules (40%) in 36 patients were confirmed as benign:
- −18 nodules were stable or reduced in size at ≥2 years;
- 6 nodules disappeared during the follow-up (disappearance within an average of 4.7 months ± 1.75 [2–6];
- 2 nodules had a benign histology (a subpleural lymph node by analysis of the excision piece and organized pneumonia after carrying out a transthoracic biopsy, which was considered representative);
- 10 nodules had typical characteristics of intrapulmonary lymph nodes.
- −13 nodules (14.4%) in 10 patients were confirmed malignant by biopsy:
- 3 squamous cell carcinomas; 3 adenocarcinomas (in 2 patients); 2 small-cell bronchial carcinomas, 3 metastases (in 1 patient) and 2 others;
- -41 nodules (45.5%) in 38 patients remained undetermined at the closing date of data retrieval:
  - 36 nodules without any follow-up;
  - And 5 nodules with insufficient follow-up (<2 years); these 5 nodules were stable over the short time period available.

### 3.3 Performance of artificial intelligence (AI)

The AI analysis was not feasible on 9 out of 90 nodules (9 patients) due to the technical issues (DICOMs not accepted by the software).

Finally, 81 nodules in 74 patients (34 confirmed benign, 11 confirmed malignant and 36 undetermined) were successfully sent to the server. All nodules were detected, segmented and analyzed automatically by the software. The mean AI score of these 81 nodules was 64.3 ± 33.7 [0.02–100].

The correlation between the nature of the nodules and the AI categorization is listed in Table 2.

**Table 2:** Correlation between the nature of the pulmonary nodules and the IA score.

	Benign nodules *N* = 34	Malignant nodules *N* = 11	Undetermined nodules *N* = 36
Categorized as benign (AI score < 50%)	16	0	7
Categorized as intermediate (AI score between 50 and 75%)	3	0	3
Categorized as malignant (AI score> 75%)	15	11	26

Among the group of analyzed nodules which were confirmed benign (*n* = 34), the nodules had an AI score between 0.02 and 96.73 (mean = 48.05 ± 37.32).

- 16 nodules were classified as benign (AI score<50%),
- 15 nodules were classified as malignant (AI score > 75%)
- And 3 nodules were classified as intermediate

Among the group of analyzed nodules which were confirmed malignant (*n* = 11), the nodules had an AI score comprised between 82.89 and 100 (mean = 93.9 ± 2.3):

- The 11 nodules were categorized as malignant (AI score > 75%)
- No nodules were categorized as benign or intermediate.

Among the group of analyzed nodules which were undetermined (*n* = 36), the nodules had a AI score between 3.93 and 97.28 (mean = 70.5 ± 26.5),

- 26 nodules were categorized as malignant ;
- 7 nodules were categorized as benign ;
- And 3 nodules were categorized as intermediate.

The diagnostic performance of AI software for the positive diagnosis of malignant nodules is as follows:

- In the event that the nodules categorized as intermediate (50–75%) by the software are recategorized as benign: sensitivity = 100% [95% CI 72%-100%]; specificity = 55.8% [38–73] ; PPV = 42.3% [23–63]; NPV = 100% [82–100].
- In the event that the nodules categorized as intermediate (50–75%) are recategorized as malignant: sensitivity = 100% [72–100] ; specificity = 47% [30–65] ; PPV = 37.9% [21–58]; NPV = 100% [79–100].

### 3.4 Performance of the Brock model

Among the 81 nodules analyzed by the software, 34 nodules were categorized benign (probability < 5%) and 47 nodules were categorized as potentially malignant (probability > 5%) following the probability of malignancy calculated by the Brock model.

The correlation between the nature of the nodules and the categorization according to the Brock model is listed in Table 3.

**Table 3:** Table of correlation between the nature of the pulmonary nodules and the Brock model, by using a threshold of 5% probability of malignancy.

	Benign nodules *N* = 34	Malignant nodules *N* = 11	Undetermined nodules *N* = 36
Categorized as benign (< 5%)	22	1	11
Categorized as potentially malignant (> 5%)	12	10	25

The performances of the Brock model for the diagnosis of malignant nodules using a threshold of 5% are as follows: sensitivity = 90.9% [59–100]; specificity = 64.7% [46–80]; PPV = 45.4% [24–68]; NPV = 95.6% [78–100].

Among the group of confirmed benign nodules (*n* = 34), the nodules had a probability of malignancy between 0.54 and 59.18% (mean = 9.8 ± 14.4);

- 22 nodules were categorized as probably benign.
- And 12 nodules were wrongly categorized as potentially malignant by the model.

Among the group of confirmed malignant nodules (*n* = 11), the nodules had a probability of malignancy between 4.98 and 62.2% (mean = 23.6 ± 18.6);

- 10 nodules were categorized as potentially malignant.
- And 1 nodule was wrongly categorized as probably benign by the model.

Among the group of undetermined nodules (*n* = 36), the nodules had a probability of malignancy between 0.81 and 63.06% (mean = 16,4 ± 16,63);

- 11 nodules were considered as benign.
- And 25 nodules were considered as potentially malignant.

### 3.5 Impact of artificial intelligence software in comparison with the radiologist's recommendations

For 50 out of 90 nodules, guidelines for the management of pulmonary nodules were written in the report by the radiologist who initially interpreted the examination.

Among the category of nodules finally confirmed benign and analyzed (*n* = 34), a recommendation for the management of the nodule was issued for 14 out of 34 nodules (20 out of 34 benign nodules had no management recommendation).

For each case, the guidelines consisted in a follow-up recommendation (for 9 nodules, the recommendation was a remote reassessment without specifying a time limit; for 4 nodules, the recommendation was a reassessment after 3 months; for 1 nodule, the recommendation was a reassessment after a time of between 6 months and 9 months).

These follow-up guidelines were finally applied for 12 out of the 14 nodules (86%), among which 6 nodules (50%) were initially categorized as benign by AI software (IA score < 50%). Therefore, the use of AI software could have made it possible to consider these 6 nodules as benign right from the initial scan.

Among the category of nodules finally confirmed malignant and analyzed (*n* = 11), a recommendation for the management of the nodule was issued for 6 out 11 nodules (5 out of 11 malignant nodules had no management recommendation). All the nodules were categorized as malignant by the software (AI score > 75%).

The recommendations for the 6 nodules were the following:

- For 5 nodules (in 4 patients), an indication of a specialist opinion, with, each time, a short diagnostic delay of malignancy (< 1 month),
- For 1 nodule, a recommendation of follow-up after 3 months, which was finally not applied, with a long diagnostic delay after 17 months. The use of AI logic could have made it possible to reduce this diagnostic delay, by highlighting the need for follow-up, given the high AI score (> 75%).

Regarding the 5 nodules without management recommendation:

- 2 nodules had a long diagnostic delay, respectively 11 months and 25 months. For these nodules, the AI ​​software could potentially have made possible to reduce this diagnostic delay by indicating a need for follow-up, given the high AI score (> 75%);
- Three nodules in the same patient (metastases) finally had a short diagnostic delay, despite the lack of a recommendation for follow-up.

The use of AI software could possibly have shortened the diagnostic delay by several months for 3 out of 11 malignant nodules (3 out of 8 patients).

## 4 Discussion

This monocentric retrospective study included 90 pulmonary nodules in 83 patients and aimed to assess the interest of a deep learning-based artificial intelligence software in characterizing pulmonary nodules detected incidentally on emergency CT examinations – outside the context of lung cancer screening. The fortuitous discovery of these pulmonary nodules is a frequent and problematic situation since the determination of their nature may require, depending on the case, follow-up scans over several years or additional explorations (PET-CT, biopsy or surgery), despite the fact that the majority of these nodules are benign. In our study population, which includes a retrospective analysis of chest scans acquired over 6 consecutive months, 83 out of 2673 patients (3%) had at least one ≥ 6 mm incidental lung nodule not already known, and 14 nodules (21% of nodules, 0.5% of initial patients) were ultimately malignant. The incidence of incidentally discovered lung nodules was lower than that described in medical literature, while their rate of malignancy was higher. This difference is possibly explained by our stricter inclusion and exclusion criteria.

Our main objective was to investigate the impact of using such software on the management of incidental lung nodules. Compared to the radiologist's recommendations (which were present in only 50% of cases, and followed in approximately 50% of cases), adding the AI score could have made it possible to avoid the follow-up of nodules which were finally benign in 50% of cases (6 out of 12 nodules), thanks to its very high NPV. However, the possible impact of speeding up the diagnosis on nodules which were finally confirmed as malignant is more debatable. Indeed, the PPV is low and false positives were common. Therefore, initiating or intensifying the management of the nodules solely based of a positive AI score doesn't seem to be a "profitable" practice.

The AI software had excellent sensitivity (100%) in detecting malignant nodules, as all confirmed malignant nodules were correctly classified as dangerous by the software. However, the specificity was low, estimated to be around 50%. To categorize the nodules, we used the manufacturer's recommendations, which suggested that nodules should be classified as benign if the score was <50%, intermediate if the score was between 50 and 75%, and dangerous if the score was >75%. This high sensitivity and low specificity may have been influenced by the exceedingly high detection threshold for malignant nodules. Adjusting this threshold could potentially improve the software's specificity, but at the risk of reducing its sensitivity.

Our secondary objective was to compare the performance of AI software with Brock's multivariate model. We chose to use a threshold of 5% of risk of malignancy to categorize the nodules using the Brock model. According to the study by McWilliams et al. [14], this threshold has a sensitivity, specificity, PPV and NPV of 71.4%, 95.5%, 18.5% and 99.6%, respectively, on lung cancer screening cohorts. In our study population, sensitivity was 90.9%, specificity was 64.7%, PPV was 45.4%, and NPV was 95.6%. Thefore the model's sensitivity was lower than that of the AI software, but it had better specificity.

Several recent studies have highlighted the potential of artificial intelligence in the diagnosis and management of indeterminate lung nodules. Massion et al. [22] demonstrated on two independent cohorts of incidental pulmonary nodules that a deep-learning algorithm was associated with better precision in the prediction of the probability of malignancy than a conventional risk calculator (Mayo model), with a significant difference statistically (ASC of 91.9% versus 81.9%, and 93.5% versus 78.1% on the two external cohorts). The model also reclassified indeterminate pulmonary nodules as low or high risk in more than 30% of cases compared to the Mayo model, potentially changing the follow-up of patients. Similarly, Venkadesh et al. [23] developed a deep-learning algorithm which significantly outperforms Brock's model (ASC of 0.93 versus 0.90) in a screening cohort (Danish Lung Cancer Screening Trial).

In another study closer to ours, which was also looking at the clinical consequences of AI software on incidental lung nodules, Tsakok et al. [24] showed that compared to clinical practice, deep-learning AI could have enabled an overall reduction in CT follow-up by 18.6% of benign nodules with a low probability score; it could also have accelerated patient care by a few months in 5 out of 10 patients with pulmonary nodules with a high probability score of malignancy.

Our study has several limitations. The main limitation concerns its monocentric and retrospective nature, as well as the relatively low number of nodules included, with a high number of nodules remaining of undetermined nature. The low number of nodules whose nature is confirmed (36 benign and 13 malignant) and high proportion of indeterminate nodules thus makes it difficult to determine the exact diagnostic performance of the software on a larger scale. Indeterminate nodules have been excluded from the performance analysis of the software and some of these nodules have been classified as benign by the artificial intelligence software (7/36). If some of these nodules was ultimately malignant, then the NPV of the software would be worse.

To conclude, we found that in a context of incidental pulmonary nodules, an artificial intelligence software using deep learning algorithms presents, on a retrospective cohort of 90 nodules, a strong NPV (100%) for malignancy on the nodules whose final nature is confirmed. In clinical practice, this algorithm could help the radiologist optimize his recommendations of follow-up, and to reduce the need of follow-up of nodules categorized as benign.

## CRediT authorship contribution statement

**Michelin Bastien:** conception, data retrieval, data analysis, validation, writing. **Aïssam Labani:** conception, data retrieval, data analysis, validation. **Pascal Bilbault:** data retrieval, data analysis, validation. **Catherine Roy:** data retrieval, data analysis, validation. **Mickaël Ohana:** conception, data retrieval, data analysis, validation, writing.

## Declaration of Competing Interest

The authors declare that they have no known competing financial interests or personal relationships that could have appeared to influence the work reported in this paper.
